# Uncovering the origin of enhanced field emission properties of rGO–MnO_2_ heterostructures: a synergistic experimental and computational investigation†

**DOI:** 10.1039/d0ra03360j

**Published:** 2020-07-10

**Authors:** Sachin R. Rondiya, Indrapal Karbhal, Chandradip D. Jadhav, Mamta P. Nasane, Thomas E. Davies, Manjusha V. Shelke, Sandesh R. Jadkar, Padmakar G. Chavan, Nelson Y. Dzade

**Affiliations:** School of Chemistry, Cardiff University Main Building, Park Place Cardiff CF10 3AT Wales UK RondiyaS@cardiff.ac.uk DzadeNY@cardiff.ac.uk; Physical and Materials Chemistry Division, CSIR-National Chemical Laboratory Pune 411008 MH India; The State Key Laboratory of Refractories and Metallurgy, Institute of Advanced Materials and Nanotechnology, College of Materials and Metallurgy, Wuhan University of Science and Technology Wuhan 430081 P. R. China; Department of Physics, Savitribai Phule Pune University Pune 411007 India; Department of Physics, School of Physical Sciences, Kavayitri Bahinabai Chaudhari North Maharashtra University Jalgaon 425001 India

## Abstract

The unique structural merits of heterostructured nanomaterials including the electronic interaction, interfacial bonding and synergistic effects make them attractive for fabricating highly efficient optoelectronic devices. Herein, we report the synthesis of MnO_2_ nanorods and a rGO/MnO_2_ nano-heterostructure using low-cost hydrothermal and modified Hummers' methods, respectively. Detailed characterization and confirmation of the structural and morphological properties are done *via* X-ray Diffraction (XRD), Field Emission Scanning Electron Microscopy (FESEM) and Transmission Electron Microscopy (TEM). Compared to the isolated MnO_2_ nanorods, the rGO/MnO_2_ nano-heterostructure exhibits impressive field emission (FE) performance in terms of the low turn-on field of 1.4 V μm^−1^ for an emission current density of 10 μA cm^−2^ and a high current density of 600 μA cm^−2^ at a relatively very low applied electric field of 3.1 V μm^−1^. The isolated MnO_2_ nanorods display a high turn-on field of 7.1 for an emission current density of 10 μA cm^−2^ and a low current density of 221 μA cm^−2^ at an applied field of 8.1 V μm^−1^. Besides the superior FE characteristics of the rGO/MnO_2_ nano-heterostructure, the emission current remains quite stable over the continuous 2 h period of measurement. The improvement of the FE characteristics of the rGO/MnO_2_ nano-heterostructure can be ascribed to the nanometric features and the lower work function (6.01 and 6.12 eV for the rGO with 8% and 16% oxygen content) compared to the isolated α-MnO_2_(100) surface (*Φ* = 7.22 eV) as predicted from complementary first-principles electronic structure calculations based on density functional theory (DFT) methods. These results suggest that an appropriate coupling of rGO with MnO_2_ nanorods would have a synergistic effect of lowering the electronic work function, resulting in a beneficial tuning of the FE characteristics.

## Abstract 2

The unique structural merits of heterostructured nanomaterials including the electronic interaction, interfacial bonding and synergistic effects make them attractive for fabricating highly efficient optoelectronic devices.

## Graphical abstract

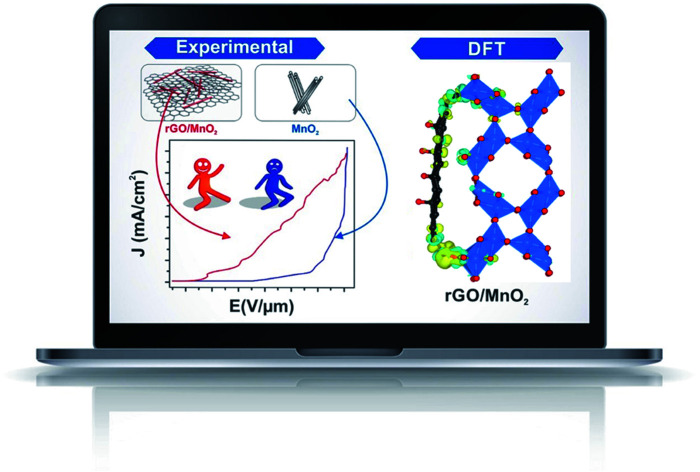

## 1. Introduction

Nanoscale heterostructure design comprising different material compositions is emerging as an attractive strategy and essential building block for functional devices to achieve improved performance. The desired physicochemical properties of the participating nanomaterials in nanostructured hybrids/composites complement each other by tuning their electronic properties to meet the requirements for the fabrication of efficient electronic devices.^1^ Reduced Graphene Oxide (rGO) is an attractive and ideal nanomaterial to be paired with another suitable semiconductor for the development of multifunctional heterostructures because of its unique electronic properties, high electrical conductivity (5 × 10^−3^ S cm^−1^), flexible structure, high aspect ratio, and high specific surface area (2630 m^2^ g^−1^).^2–5^ Owing to its unique physicochemical properties, rGO is being recognized as a material of great interest for potential applications in nanoelectronics,^6^ nanoelectromechanical systems,^7^ sensors,^8^ catalysis,^9^ energy storage devices,^10,11^ optics,^12^ and field emission (FE).^13–15^ There exist several reports of the successful synthesis of rGO or modified graphene heterostructures with various semiconducting nanomaterials such as TiO_2_, SnO_2_, ZnO, Si, CdSe, *etc.* in the literature.^16–18^

For field emission applications, where electrons are extracted from the surface of a metal/semiconductor by an electrostatic field through quantum mechanical tunneling, rGO-based nanocomposites such are rGO-Bi_2_S^19^ and WS_2_-RGO,^20^ have demonstrated superior field emission properties. Among transition metal oxides, manganese dioxide (MnO_2_) has attracted increasing interest for field emission applications, owing to their wide structural diversity combined with unique chemical and physical properties.^21,22^ The advantages of MnO_2_ as field emitter are the lower cost for raw materials and the fact that manganese is more environmentally friendly than other metal oxide.^23,24^ MnO_2_ has also attracted a lot of attention as an electrochemical pseudocapacitor material due to its high theoretical capacitance (1370 F g^−1^).^25–27^ Wu *et al.* reported inspiring results such as low turn-on field value of 8.4 V μm^−1^ at current density of 1 μA cm^−2^ and maximum emission current density of 160 μA cm^−2^ at an applied field 18 V μm^−1^.^21^ The field emission applications of MnO_2_ is, however, limited by its low specific surface area and poor electrical conductivity (10^−5^ to 10^−6^ S cm^−1^). Compared to its flat films, by fabricating rGO/MnO_2_ nanocomposite the interface area can be significantly enlarged, which is desirable for field emission application.^28^ Besides, rGO is solution-processable and thus can be deposited in large areas onto different kinds of substrates enabling simple and cost-effective fabrication of field electron emitters for display applications. The formation of rGO–MnO_2_ nanostructures and their electrochemical performance have been extensively investigated and it was demonstrated that compared to the single metal-oxide, rGO/MnO_2_ nanocomposites show superior electric conductivity, electric capacity and charge/discharge efficiency for supercapacitor performance.^29–34^ These characteristics make rGO/metal-oxide nanocomposites promising materials for energy applications. Considering that field emission is geometry (shape, size, aspect ratio, alignment, and areal density of the nanostructure) and work function dependent phenomenon, well-aligned rGO/MnO_2_ nanostructures is promising for enhancement of field emission characteristics.^35^

Herein, we report a simple and cost-effective solution-based method to prepared MnO_2_ nanorods and rGO/MnO_2_ nano-heterostructure. The structural and morphological verifications have been done by using X-ray diffraction (XRD), Field Emission Scanning Electron Microscope (FESEM), and Transmission Electron Microscopy (TEM). Finally, the field emission properties of the as-prepared MnO_2_ nanorods and rGO/MnO_2_ nano-heterostructure was systematically characterized and compared. The rGO/MnO_2_ nano-heterostructure exhibits superior field emission characteristics compared to the MnO_2_ nanorod. The rGO/MnO_2_ nano-heterostructure demonstrates a low turn-on field of 1.4 V μm^−1^ for an emission current density of 10 μA cm^−2^ compared to 7.1 V μm^−1^ for MnO_2_ nanorod. The combined contribution of the sharp edges of the thin rGO sheets and high aspect ratio of the MnO_2_ nanorods, coupled with synergetic effect in the rGO/MnO_2_ nano-heterostructure are responsible for the observed enhanced field emission behavior. Consistent with the experimental data, our complementary first-principles DFT calculations predict lower work function for the rGO/MnO_2_ nano-heterostructure compared to the isolated MnO_2_ as the primary origin for improved field emission.

## 2. Experimental, characterization, and computational methods

### 2.1 Synthesis of MnO_2_ and rGO/MnO_2_

**Scheme 1:**
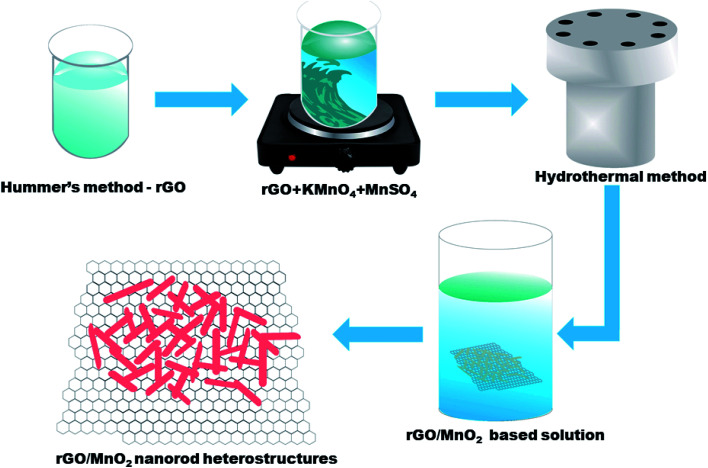
The facile synthesis of MnO_2_ nanorods and rGO/MnO_2_ heterostructure.

The rGO has been synthesized by modified Hummer's method^36^ whereas the MnO_2_ nanorods has been synthesized by the hydrothermal method.^37^ For the preparation of the rGO/MnO_2_ composite, 1 mg ml^−1^ rGO was dispersed in the 100 ml of DI water in a beaker. Later, 10 mM of KMnO_4_ and 10 mM of MnSO_4_ were added into the rGO solution and stirred for 30 min form a homogenous solution. The prepared solution was transferred into a stainless steel autoclave and kept at 160 °C for 24 h. After cooling to room temperature, the material was filter washed with DI water and ethanol to obtian rGO/MnO_2_ composite, which was dried in an oven at 80 °C for 12 hours and used for various characterizations presented next. Scheme 1 represents the synthesis steps which were followed for synthesis of rGO, MnO_2_ and rGO/MnO_2_ heterostructure.

### 2.2 Materials characterization

The MnO_2_ nanorods and rGO/MnO_2_ nano-heterostructure were characterized by various complementary experimental methods. The XRD patterns were obtained with a Bruker D8 Advance X-ray diffractometer using the Cu Kα line (*λ* = 1.54 Å) at 1° grazing angle. The HR-TEM micrographs and selected area electron diffraction (SAED) patterns were obtained with JEOL-JEM 2100 microscope operating at 200 kV. The samples were dry dispersed over 300 mesh copper grids coated with holey carbon film. A Field Emission Scanning Electron Microscope (FEG-SEM Model – Tescan MAIA3) was used to examine the morphology and surface topography of the MnO_2_ nanorods and rGOs/MnO_2_ composite. The accelerating voltage was 15 kV. X-ray spectroscopy (EDS) measurements were done using Oxford Instruments X-Max^N^ 80 detector and analyzed using Aztec software. X-ray Photoelectron Spectroscopy (XPS) was carried on the samples using a Kratos Axis Ultra DLD photoelectron spectrometer utilizing monochromatic AlKα radiation operating at an energy of 120 W (10 × 12 kV). Data were analyzed using Casa XPS and modified Wagner sensitivity factors as supplied by the instrument manufacturer after subtraction of a Shirley background. All spectra were calibrated to the C(1s) line taken to be 284.8 eV.

### 2.3 Field emission

The field emission studies of the MnO_2_ nanorods and rGO/MnO_2_ heterostructure were carried out in the Ultra-High Vacuum (UHV) chamber at a base pressure of ∼1 × 10^−8^ mbar (Excel Instruments model: I-100). Detail experimental procedure may found in our earlier paper.^38^ The configuration of the field emission experiment steps up is shown in ESI (scheme S1).† The distance between inter-electrode was maintained at 1 mm. The area of both specimens (MnO_2_ nanorods and rGOs/MnO_2_heterostructure) was 0.25 cm^2^.

### 2.4 Computational methods

The first-principles spin polarized density functional theory (DFT) calculations were performed using the Vienna *Ab initio* Simulation Package (VASP),^39–41^ a periodic plane wave DFT code which includes the interactions between the core and valence elections using the Project Augmented Wave (PAW) method.^42^ An energy cut-off of 600 eV, and Monkhorst–Pack^43^*k*-point mesh of 7 × 7 × 3 was used to sample the sample the Brillouin zone of bulk α-MnO_2_. Geometry optimizations were performed based on the conjugate-gradient algorithm until the residual Hellmann–Feynman forces on all relaxed atoms reached 10^−3^ eV Å^−1^. The electronic exchange–correlation potential was calculated using the Perdew–Burke–Ernzerhof (PBE) generalized gradient approximation (GGA) functional.^44^ To accurately reproduce the experimentally known band gaps and density of states features of α-MnO_2_ and rGO, the screened hybrid functional HSE06 ^45^ was used with the exchange value of 25%. The projected density of states (PDOS) was calculated using tetrahedron method with Bloch correction.^46^

The most stable α-MnO_2_ (100) surface^47^ was employed to form the nano-heterostructure with rGO (rGO/α-MnO_2_). The α-MnO_2_ (100) surface was created from the optimized bulk material using the METADISE code, which ensures the creation of surfaces with zero dipole moment perpendicular to the surface plane. The rGO/α-MnO_2_nano-heterostructure was constructed with (2 × 4)-α-MnO_2_(100) and (5 × 5)-rGO supercells. We used *k*-point meshes of 9 × 9 × 1 for the rGO monolayer, 5 × 5 × 1 for the α-MnO_2_ (100) surface, and 5 × 5 × 1 for the rGO/α-MnO_2_ composite. In each simulation cell, a vacuum region of length 20 Å was added perpendicular to the surface to avoid interactions between periodic slabs. The electrostatic potential of each surface was averaged along the *c*-direction, using the Macro Density package.^48–50^ The work function (*Φ*) was calculated as *Φ* = *V*_vacuum_ − *E*_F_, where *V*_vacuum_ and *E*_F_ are the vacuum and Fermi level, respectively. Dipole correction perpendicular to all surfaces was accounted for, which ensured that there is no net dipole perpendicular to the surfaces that may affect the potential in the vacuum level.^51–53^

## 3. Results and discussions

### 3.1 Characterization of MnO_2_ and rGO/MnO_2_

**Fig. 1:**
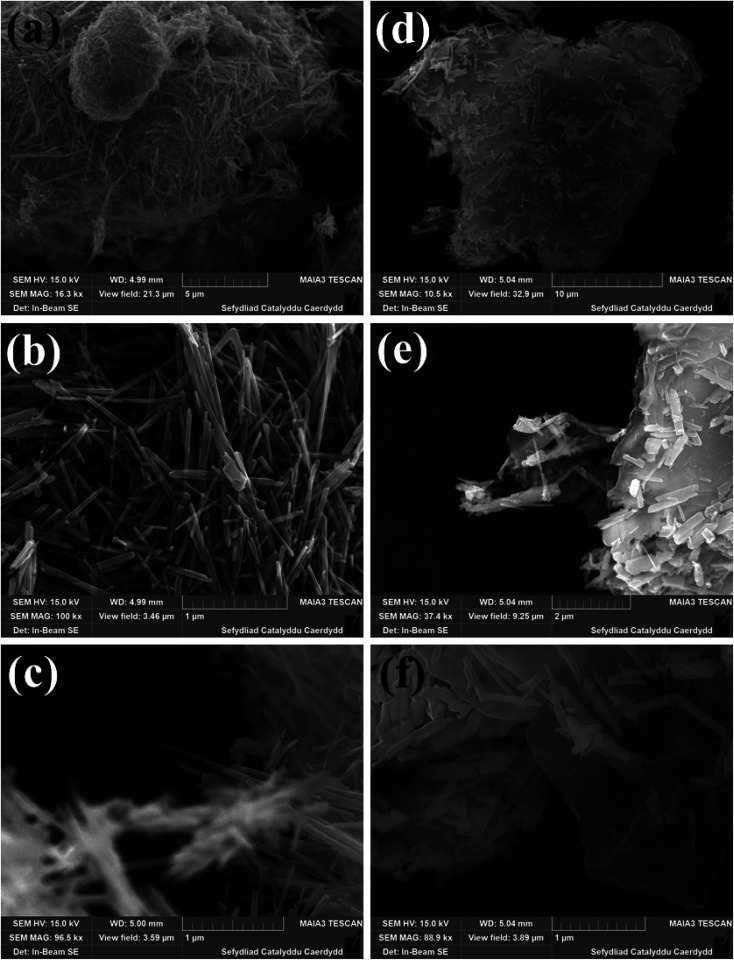
FESEM images of MnO_2_ nanorods (a–c) and rGO–MnO_2_ heterostructures (d–f) recorded at different magnification.

The crystalline structures of the MnO_2_ nanorods and rGO/MnO_2_ nano-heterostructure were confirmed by XRD and the corresponding results are presented in Fig. S1.† All the diffraction peaks in Fig. S1† can be indexed to the tetragonal crystal structure of MnO_2_ (ICDD card no. 72-1982) with lattice constant *a* = *b* = 9.815 Å and *c* = 2.847 Å. We have observed the highest growth of α-MnO_2_ in the (211) plane.^54^ The XRD diffraction pattern of the rGO–MnO_2_ nanostructure is shown in inset of Fig. S1.† The broad peak at 2*θ* around 26° corresponds to the (002) plane of the reduced graphene oxide.^55^ The field emission scanning electron microscope (FESEM) images in Fig. 1, reveal the morphological properties of the MnO_2_ nanorods and rGO/MnO_2_ nano-heterostructure. The FESEM images recorded at different magnifications (panels a–c of Fig. 1) show the formation of randomly distributed MnO_2_ nanorods. The low magnification image shown in Fig. 1a depicts large coverage of the MnO_2_ nanorods. The diameter of the formed ultra-long nanorods is estimated in the range of 140–150 nm as revealed by the high magnification FESEM images analyses (Fig. 1b and c). It is evident from the FESEM image of the rGO/MnO_2_ nano-heterostructure that the MnO_2_ nanorods are embedded in the rGO network (Fig. 1b–d). The high magnification image in Fig. 1f reveals the enormous coverage of the rGO/MnO_2_ nano-heterostructure.

**Fig. 2:**
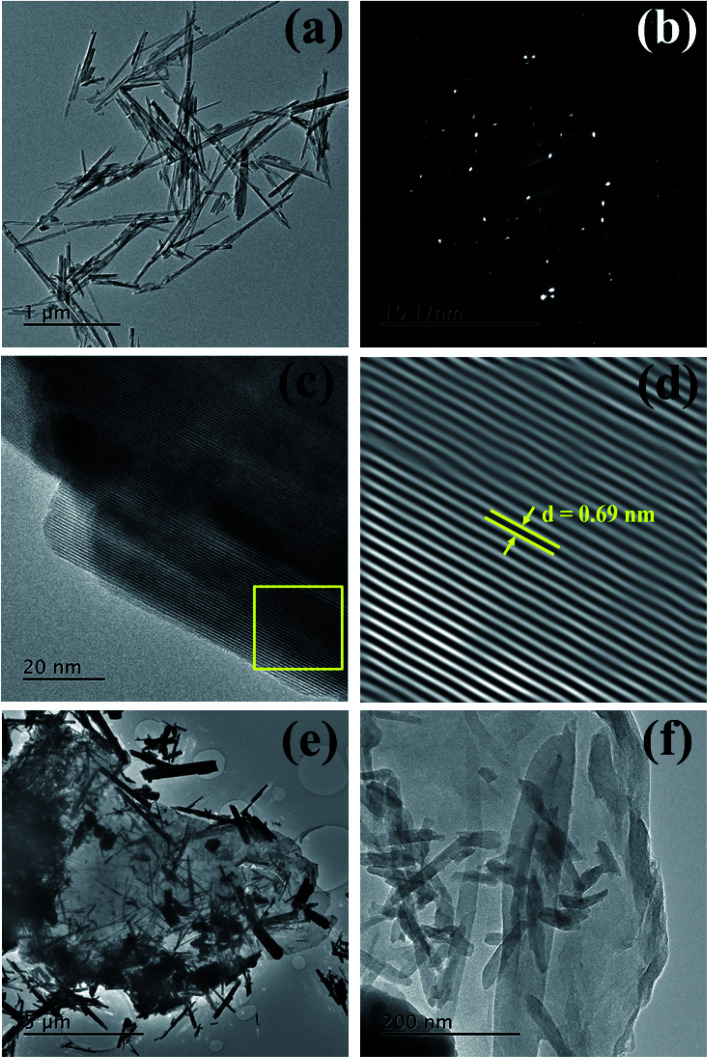
(a) TEM images of MnO_2_ nanorods (b) selected area electron diffraction (SAED) pattern of MnO_2_ nanorods (c) HR-TEM image of MnO_2_ nanorods with clear lattice resolution (d) Inverse Fourier transform of area shown in (c) with interplanar spacing of MnO_2_ (e and f) TEM images of rGO–MnO_2_ heterostructures.

Energy dispersive X-ray spectroscopy (EDX) composition analysis in the 0–10 keV energy range (ESI, Fig. S2†) confirmed that the MnO_2_ nanorods and rGO/MnO_2_ nano-heterostructure have the optimal stoichiometric atomic Mn : O and Mn : O : C ratios, respectively. Moreover, the FESEM-EDS elemental mapping (ESI, Fig. S2†) confirms an even distribution of the chemical constituents (Mn, O, and C). For detail structural analysis of the as-synthesized MnO_2_ nanorods and rGO/MnO_2_ nano-heterostructure, TEM studies were carried out. The TEM micrograph (Fig. 2a) reveals the morphology of the MnO_2_ nanorods, with sizes ranging between 50 and 70 nm in width and the average length of 1 μm. The selected area electron diffraction (SAED) pattern, depicted in Fig. 2b, confirms the polycrystalline nature of the MnO_2_ nanorods. The lattice-resolved high-resolution transmission electron microscopy (HRTEM) image of the MnO_2_ nanorod (Fig. 2c) clearly reveals its crystalline nature. The lattice fringes are clearly observed in the HR-TEM image (Fig. 2c and d) and a 0.69 nm interplanar distance indicates these planes to be of the (110) character.^56^ The inverse FFT HR-TEM image and the corresponding profile plot of the MnO_2_ nanorod are shown in Fig. S3(a and b).† A number of firmly attached α-MnO_2_ nanorods onto the rGO sheets can be clearly seen from Fig. 2e and f. X-ray photoelectron spectroscopy (XPS) analysis was applied to determine the oxidation state and elemental composition of the prepared MnO_2_ nanorods and rGO/MnO_2_ nano-heterostructure. The XPS results for the MnO_2_ nanorods are shown in Fig. S4(a and b)† and the rGO/MnO_2_ nano-heterostructure in Fig. S4(c–e).† The peaks centered at 642.63 and 654.53 eV in the high-resolution spectrum of Mn 2p (Fig. S4a†) can be assigned to the Mn 2p_3/2_ and Mn 2p_1/2_ peaks, respectively, confirming the presence of MnO_2_.^57–60^ The deconvolution peaks of the O 1s spectrum (Fig. S4b†) can be divided into three peaks which correspond to O–Mn bonding. The high-resolution spectrum of Mn 2p and O 1s shown in Fig. S4c and S4d,† respectively, for rGO/MnO_2_ nano-heterostructure, confirm the presence of MnO_2_, whereas the C (1s) spectra (Fig. S4e†) exhibit peaks that originates from the rGO sheets.

### 3.2 Field emission investigations

**Fig. 3:**
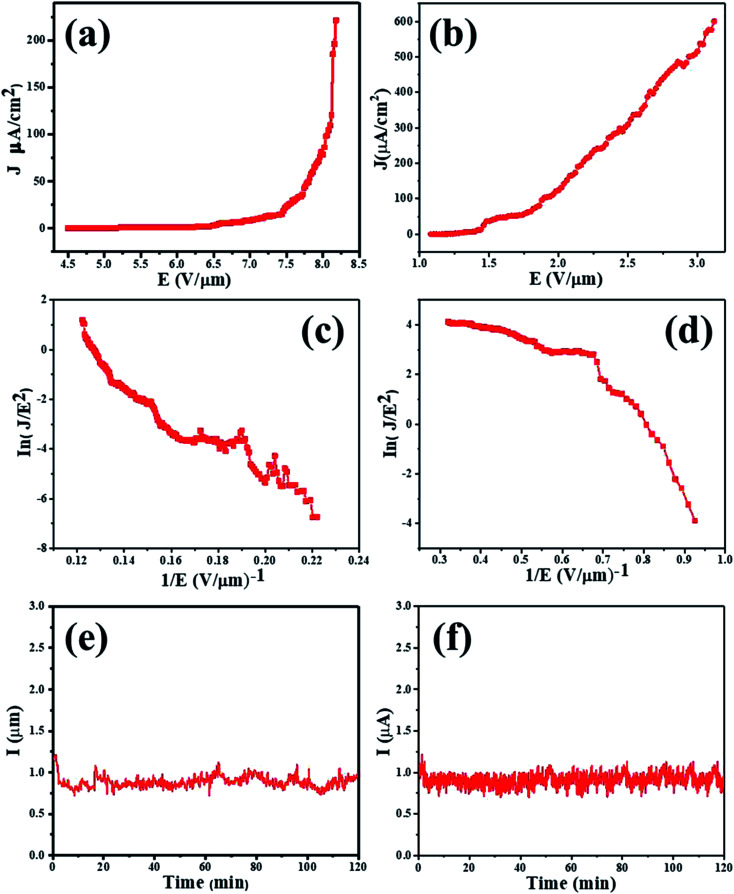
FE characteristics of the MnO_2_ nanorods and rGO–MnO_2_ heterostructures: (a and b) emission current density *versus* applied electric field (*J*–*E*) curve, (c and d) F–N plot, and (e and f) emission current *versus* time (*I*–*t*) plot.

Fig. 3(a and b) shows the emission current density as a function of the applied electrical field (*J*–*E* curves) for the MnO_2_ nanorods and rGO/MnO_2_ nano-heterostructure. In this work, the turn-on field defined as the field required to draw an emission current density (*J*) of 10 μA cm^−2^ is found to be 7.1 and 1.4 V μm^−1^ for the MnO_2_ nanorods and the rGO/MnO_2_ nano-heterostructure, respectively. The rGO/MnO_2_ nano-heterostructure also attains an impressive current density of 600 μA cm^−2^ at an applied field of 3.1 V μm^−1^ compared to the isolated MnO_2_ which displayed a lower current density of 221 μA cm^−2^ at a relatively higher applied field of 8.1 V μm^−1^. A comparison between the turn-on field values obtained in the present study and previously reported MnO_2_ nanostructures and the MnO_2_/rGO nano-heterostructures is provided in Table 1.^21,22,61–66^

**Table 1:** Comparison of the turn-on field with reported MnO_2_ nanomaterials and the MnO_2_/rGO nano-heterostructures in the present study

Sr. no.	Specimen	Turn-on field (V μm^−1^) (for *J* = 10 μA cm^−2^)	Reference
1	MnO_2_ nanotubes	8.4 (1 μA cm^−2^)	21
2	MnO_2_ nanorods	5.90 (1 μA cm^−2^)	22
3	MnO_2_/rGO nanocomposite	3.6 (1 μA cm^−2^)	
4	rGO/TiO_2_ nanocomposite	2.6	61
5	T-ZnO/rGO nanocomposite	1.54 (1 μA cm^−2^)	62
6	SnO_2_/rGO nanocomposite	1.8 (1 μA cm^−2^)	63
7	ZnO/graphene nanocomposite	1.3 (1 μA cm^−2^)	64
8	ZnO/graphene nanocomposite	2.1	65
9	SnO_2_/graphene nanocomposite	3.85 (1 μA cm^−2^)	66
10	MnO_2_ nanorods	7.1	Present work
11	MnO_2_/rGO nano-heterostructure	1.4	

The Fowler–Nordheim (F–N) plot for MnO_2_ nanorods and rGO/MnO_2_ nano-heterostructure obtained from ln(*J*/*E*^2^) verses (1/*E*) is shown in Fig. 3(c and d). Consistent previous reports,^21,22^ the FN plots of the MnO_2_ nanorods and rGO/MnO_2_ nano-heterostructure exhibit linear behavior in good agreement with their semiconducting nature. The nanometric features of the rGO/MnO_2_ nano-heterostructure coupled with the high electrical conductivity of rGO are suggested as the key factors behind the observed superior field emission properties.^2–5^ Apart from the improved field emission performance, the electron emission current stability is an important parameter for device fabrication considerations. We have therefore measured the emission current as a function of time in order to ascertain the robustness of the rGO/MnO_2_ nano-heterostructure. The emission current *versus* time (*I*–*t*) plot of the MnO_2_ nanorods and rGO/MnO_2_ nano-heterostructure were recorded continuously for 2 h at a preset value of emission current of ∼1 μA as shown in Fig. 3(e and f). Generally, the results show that the emission current remains quite stable without showing any sign of diminishing over the 2 h period of continuous testing. Instabilities in the form of spikes can be attributed to the presence of residual gas molecules across the emitter surface.

### 3.3 Density functional theory analyses

**Fig. 4:**
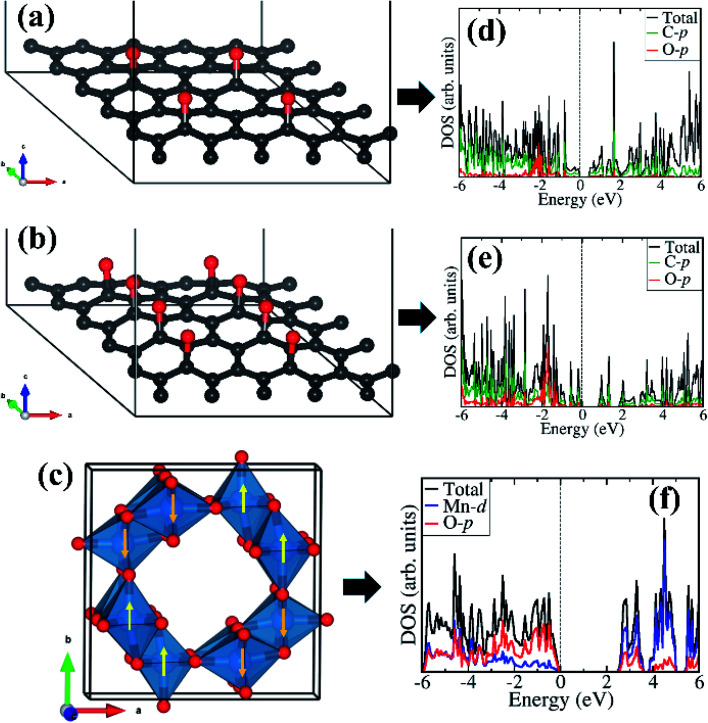
Optimized structure of rGO with epoxide functional group with (a) 8% and (b) 16% oxygen content. The tetragonal structure of α-MnO_2_ showing the AFM spin ordering in (c). The corresponding partial density of states are shown in the right column (d–f). Atomic color: C = grey, O = oxygen, Mn = blue.

Considering that field emission is a geometry and work function (*Φ*) dependent phenomenon, with lower *Φ* enhancing the field emission characteristics, we have carried out first-principles density functional theory calculations to gain atomic-level insight into the electronic structure and work function of the isolated rGO, α-MnO_2_ (100) surface, and the rGO/α-MnO_2_ (100) nanocomposite. As the electronic band gap of rGO vary depending on the degree of reduction, the rGO monolayer with two concentrations of epoxide functional groups with 8% and 16% oxygen contents were modelled as shown in Fig. 4(a and b). The band gap of the rGO with 8% and 16% oxygen contents are predicted at 0.48 eV (Fig. 4d) and 0.81 eV (Fig. 4e). This is consistent with experimental data that showed that the band gap of rGO can be tuned from 0.264–0.786 eV by controlling the surface concentration of epoxide groups.^67^ The bulk α-MnO_2_ was modelled tetragonal crystal structure (space group – *I*4/*m*, no. 87) in the antiferromagnetic AFM-C2 configuration^68^ as shown in Fig. 4c. A full unit cell relaxation yielded a strain-free α-MnO_2_ with lattice parameters *a* = *b* = 9.763 Å, *c* = 2.872 Å, which compares closely with known experimental data (*a* = *b* = 9.71 Å and *c* = 2.88 Å).^69,70^ The electronic band gap of α-MnO_2_ is predicted at 2.42 eV (Fig. 4f), in close agreement with an experimental estimate of 2.23 eV^71^ and previous theoretical prediction of 2.7 eV.^72^ The valence band edge of α-MnO_2_ is demonstrated to consist mainly of O-p states whereas the conduction band edge is dominated by Mn-d states. The smaller band gap of the rGO compared to the MnO_2_ suggests that the rGO has better electrical conductivity than the metal oxide.

**Fig. 5:**
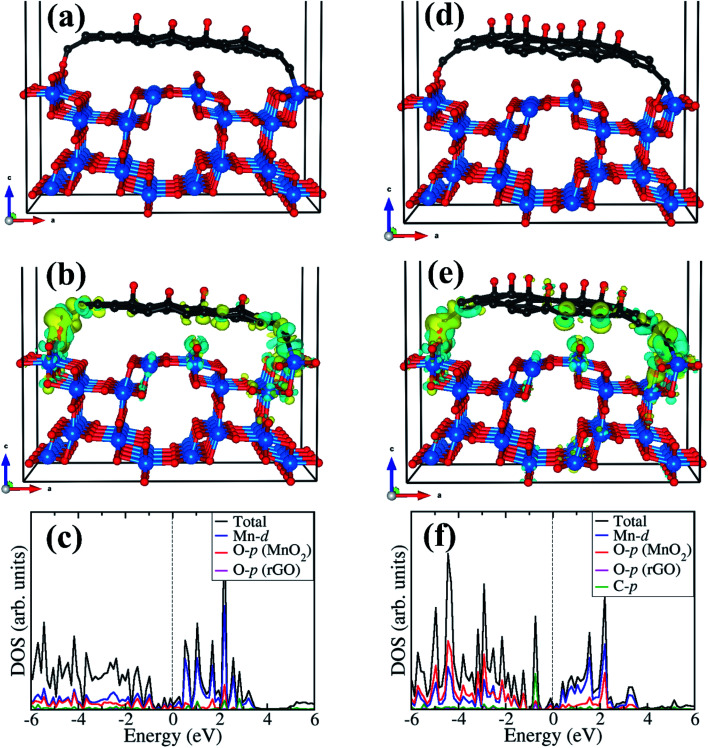
Optimized structures of the rGO/α-MnO_2_(100) nanocomposite formed by rGO with epoxide functional group (a) 8% and (d) 16% oxygen content. The corresponding differential charge density iso surface contours and partial density of states are shown in (b) and (c) for 8% oxygen content, and in (e) and (f) for 16% oxygen content. The yellow and cyan regions indicate electron density depletion and accumulation by 0.003 e Å^−3^, respectively. Atomic color: C = grey, O = oxygen, Mn = blue.

The optimized structures of the rGO/α-MnO_2_(100) nanocomposite formed by rGO with epoxide functional group with 8% and 16% oxygen contents are shown in Fig. 5a and d, respectively. The rGO is stabilized on the MnO_2_ surface *via* C–O and C–Mn chemical bonds through the terminal C atoms. The interactions of the rGO with the MnO_2_ surface gave rise to electron density redistribution within the rGO/α-MnO_2_(100) nanocomposite, which was analyzed by determining the three-dimensional-charge density difference iso surface contours as shown in Fig. 5b for the rGO with 8% oxygen content and Fig. 5e for rGO with 16% oxygen content. The yellow and cyan regions represent charge depletion and accumulation in the space, respectively. We observe electron density accumulations mainly in the interfacial bonding regions in the rGO/α-MnO_2_(100) nanocomposite, suggesting strong interactions between rGO and MnO_2_(100) surface. Consistent with the strong interaction, we observe strong hybridization between the C-p orbitals of the rGO with the Mn-d and O-p of the MnO_2_ surface as shown in Fig. 5c and f, resulting in metallic conductivity of the composite systems.

**Fig. 6:**
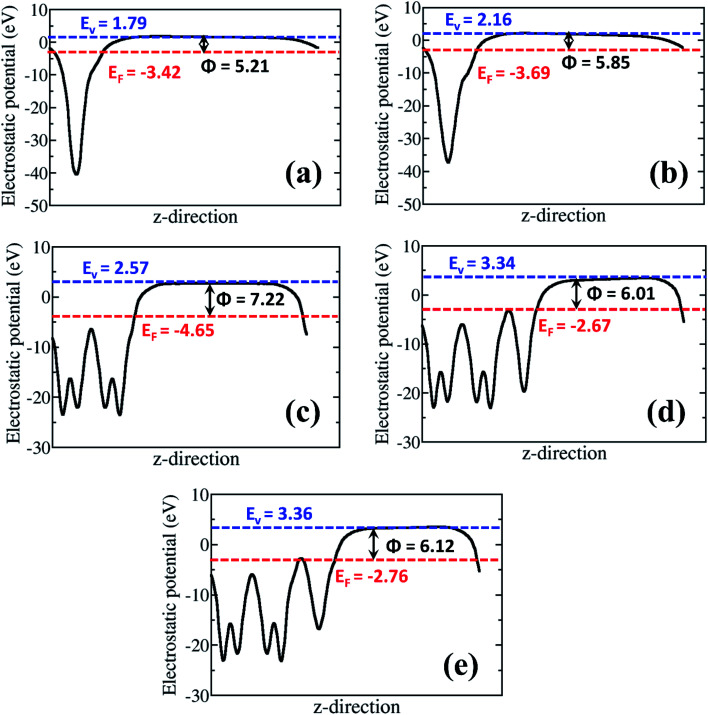
The electrostatic potentials for isolated epoxide-rGO with (a) 8% and (b) 16% oxygen content; (c) isolated α-MnO_2_(100) surface, and rGO/α-MnO_2_(100) nanocomposite formed rGO with (d) 8% and (e) 16% oxygen content. The blue and red dashed lines represent the vacuum level (*E*_vac_) and the Fermi level (*E*_F_), respectively. *Φ* denotes the work function.

The analysis of the work function (*Φ*) for the rGO monolayer, α-MnO_2_(100) and the rGO/α-MnO_2_(100) nanocomposite can help us to understand the origin/direction of charge transfer at the rGO/α-MnO_2_(100) interface. The *Φ* for the isolated rGO is predicted at 5.21 and 5.85 eV for the 8% and 16% oxygen contents, respectively (Fig. 6a and b). This is consistent with a previous theoretical investigation which predicted a work function of rGO with epoxy groups to be 4.35 eV for 1.5% oxygen content and 5.6 eV for 20% oxygen content.^73^ The *Φ* for the α-MnO_2_(100) surface is predicted at 7.22 eV (Fig. 6c), also in good agreement with earlier theoretical pH-corrected work function of 7.7 eV for the α-MnO_2_ (110) surface.^72^ The work function of the rGO/α-MnO_2_(100) nanocomposite is predicted at 6.01 and 6.12 eV for the rGO with 8% and 16% oxygen contents (Fig. 6d and e), both of which are lower than that of the isolated α-MnO_2_(100) surface (Fig. 6c). The reduction in the work function of rGO/α-MnO_2_(100) nanocomposite relative to the isolated α-MnO_2_(100) surface can be ascribed to the interfacial bonding, electronic interaction and synergistic effects. Considering that the electron emission capability of a material is dictated by its work function, the observed superior field emission characteristics of the rGO/α-MnO_2_ nanocomposite compared to the isolated α-MnO_2_ material can be attributed to the predicted lower work function for the rGO/α-MnO_2_(100) nanocomposite. Reduction in the work function has been observed in other composite materials compared to the isolated materials.^52,53^ For instance, Susaki *et al.*, have shown that the deposition of a single unit cell of MgO on an Nb:SrTiO_3_ substrate reduces the work function by about 0.8 eV.^74^ Similarly, by decorating SnSe nanosheets with Au nanoparticles (Au/SnSe) and porous ZnO nanosheets with CuSCN nanocoins (CuSCN/ZnO) resulted in significant improvements in the FE characteristics owing to predicted lower functions.^52,53^ The higher work function predicted for the α-MnO_2_(100) surface compared to rGO monolayer (Fig. 6) suggests that spontaneous electrons transfer will flow from the rGO monolayer to the α-MnO_2_(100) after the two are coupled together. Besides the reduction of the work function, the formation of nano-protrusions (denoted by red circles) in the rGO/MnO_2_ nano-heterostructure (Fig. S5†) can act as effective emission sites. In addition, the better electrical conductivity (5 × 10^−3^ S cm^−1^) of rGO is expected to plays an important role in electron transportation. The rGO as backbone may results in easy and high percolation of electrons from the rGO to MnO_2_ nanorods giving rise to the observed superior field emission behavior of the rGO/MnO_2_ nano-heterostructure.

## 4. Conclusion

In summary, we report the successful synthesis of MnO_2_ nanorods and rGO/MnO_2_ nano-heterostructure using cost effective hydrothermal and modified Hummer's methods, respectively. The coupling of rGO sheets with MnO_2_ nanorods is demonstrated to have a synergistic effect in improving the FE characteristics of formed rGO/MnO_2_ nano-heterostructure. The dramatic reduction of the turn-on field by 5.7 V μm^−1^ for an emission current density of 10 μA cm^−2^ and the achieved high current density of 600 μA cm^−2^ with an applied field of 3.1 V μm^−1^ demonstrate the superior FE characteristics of the rGO/MnO_2_ nano-heterostructure compared to the isolated porous MnO_2_ nanorods. The results are corroborated by first principles DFT calculations, which predict lower work function for the rGO/MnO_2_ nano-heterostructure (6.01 and 6.12 eV for the rGO with 8% and 16% oxygen contents, respectively) compared to the isolated MnO_2_ (7.22 eV) as the primary origin for the improved field emission of the rGO/MnO_2_ nanocomposite. The controlled nanofabrication of rGO/MnO_2_ heterostructure reported here provides a promising approach for designing highly efficient MnO_2_-based next generation FE electron sources and extend their practical applications in micro/nano electronic devices.

## Conflicts of interest

There are no conflicts to declare.

## Supplementary Material

RA-010-D0RA03360J-s001
