# Supplementary material for: Uncovering the origin of enhanced field emission properties of rGO–MnO2 heterostructures: a synergistic experimental and computational investigation
Source: RSC Adv. 2020 Jul 10;10(43):25988–98. doi: 10.1039/d0ra03360j (PMC9055343; doi:10.1039/d0ra03360j)

Electronic Supplementary Material (ESI) for RSC Advances.
This journal is © The Royal Society of Chemistry 2020

Uncovering the Origin of Enhanced Field Emission Properties of rGO-MnO_2 Heterostructures: A Synergistic Experimental and Computational Investigation

Sachin R. Rondiya[§*], Indrapal Karbhal[‡], Chandradip D. Jadhav[†], Mamta P. Nasane[‖], Thomas E. Davies[§], Manjusha V. Shelke[‡], Sandesh R. Jadkar[‖], Padmakar G. Chavan[¥], Nelson Y. Dzade[§*]

[§]School of Chemistry, Cardiff University, Main Building, Park Place, Cardiff, CF10 3AT, Wales, United Kingdom.
[‡]Physical and Materials Chemistry Division, CSIR-National Chemical Laboratory, Pune 411008, MH, India.
[†]The State Key Laboratory of Refractories and Metallurgy, Institute of Advanced Materials and Nanotechnology, College of Materials and Metallurgy, Wuhan University of Science and Technology, Wuhan 430081, P. R. China.
[‖]Department of Physics, Savitribai Phule Pune University, Pune 411007, India
[¥]Department of Physics, School of Physical Sciences, Kavayitri Bahinabai Chaudhari North Maharashtra University, Jalgaon 425001, India.

Figure S1: X-ray diffraction patterns of MnO_2 nanorods and rGO-MnO_2 heterostructure (shown in the inset).

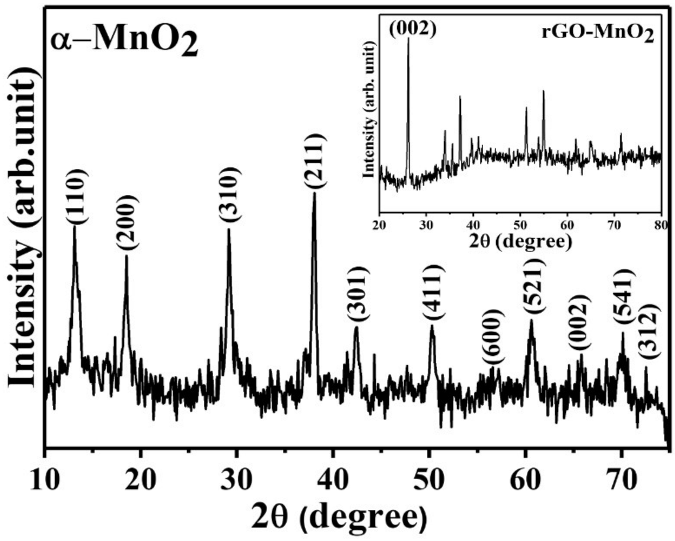

Figure S2: The EDS data and the elemental mapping suggest even distribution of chemical constituents in (a) MnO_2 nanorods and (b) rGO-MnO_2 heterostructure.

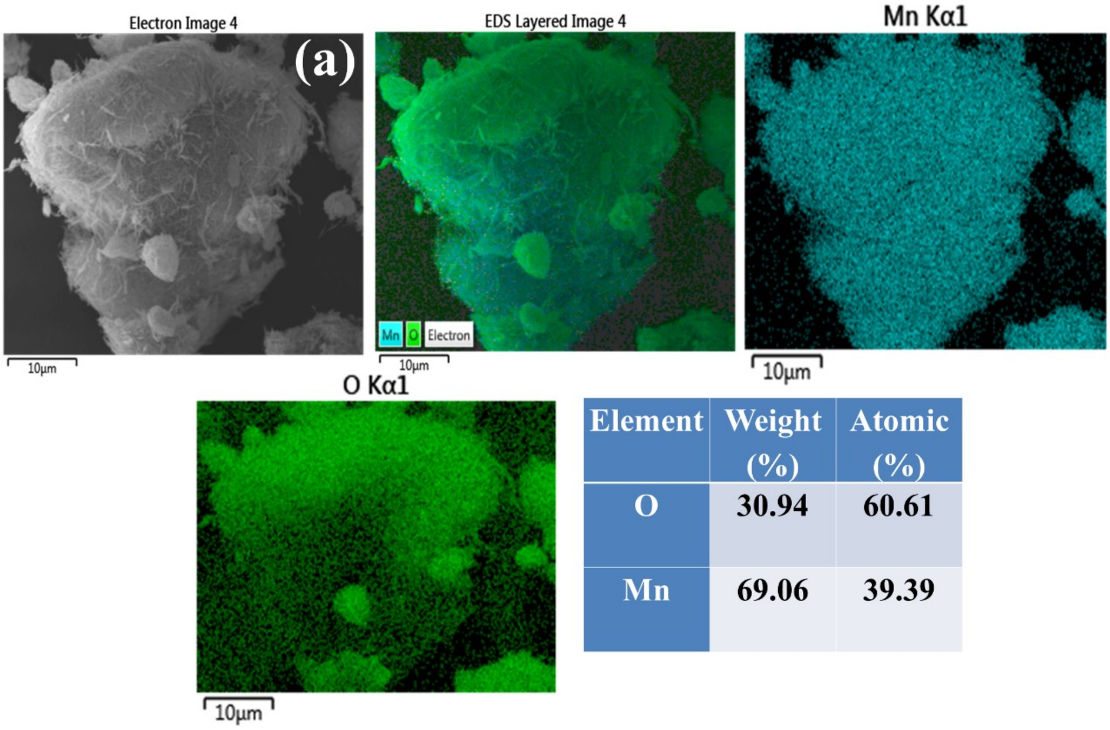

Element	Weight (%)	Atomic (%)
O	30.94	60.61
Mn	69.06	39.39

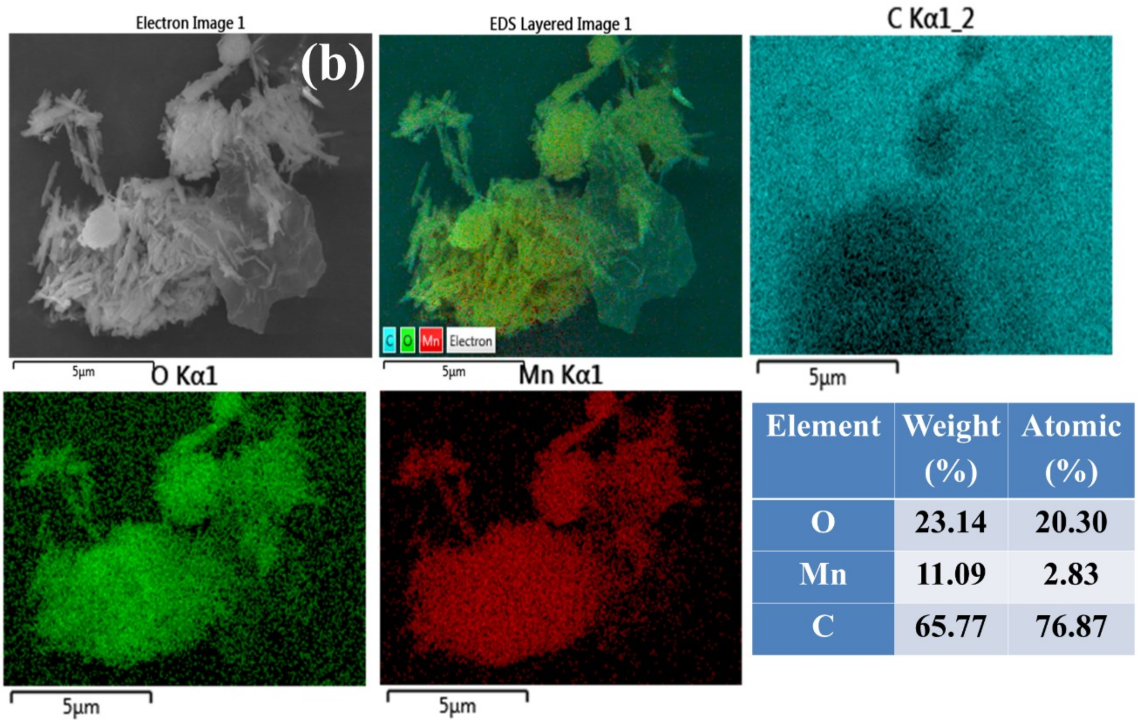

Element	Weight (%)	Atomic (%)
O	23.14	20.30
Mn	11.09	2.83
C	65.77	76.87

Figure S3: (a) HR-TEM image of MnO_2 nanorods with clear lattice resolution (d) HRTEM with interplanar spacing of MnO_2

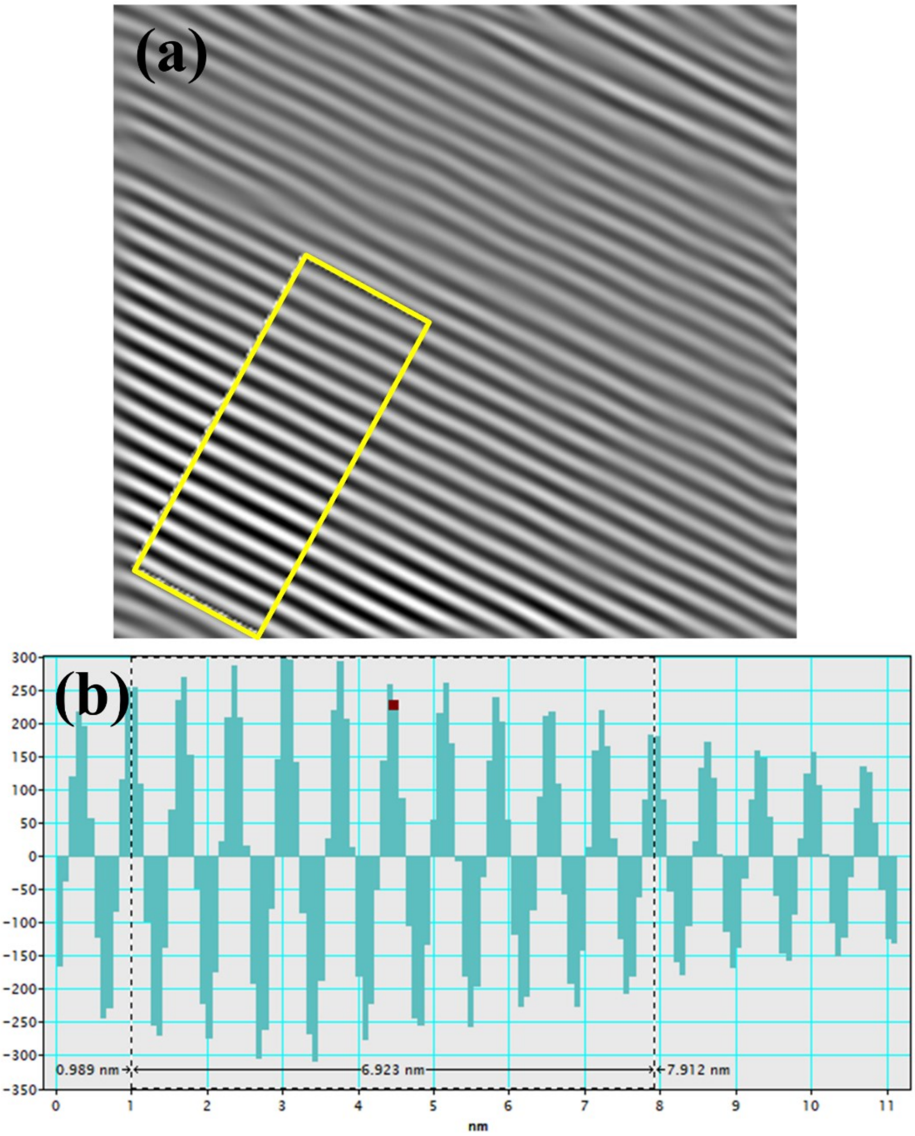

Figure S4: High-resolution XPS spectrum of (a-b) MnO_2 nanorods and (c-e) rGO-MnO_2 heterostructure.

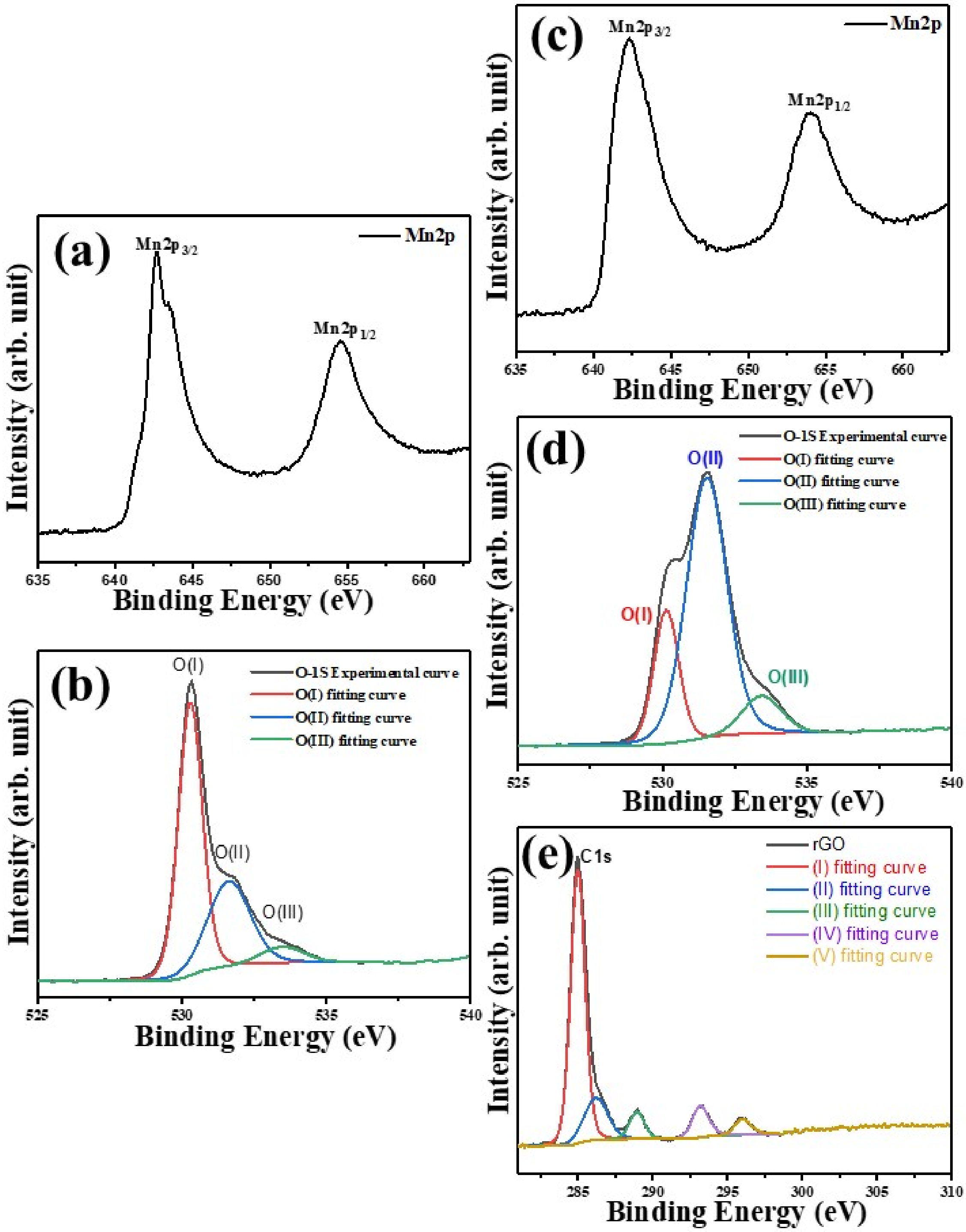

Figure S5: FESEM image of rGO/MnO2 nanoheterostructure film indicating the formation of nanoprotrusions.

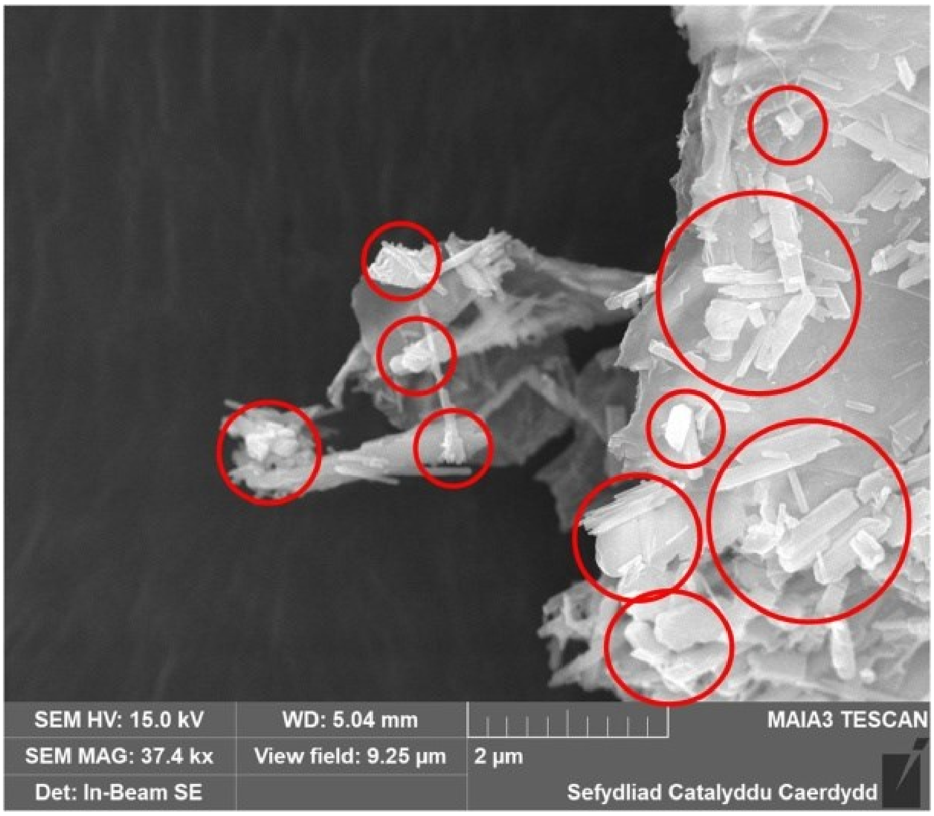

Scheme S1: The configuration of field emission setup geometry

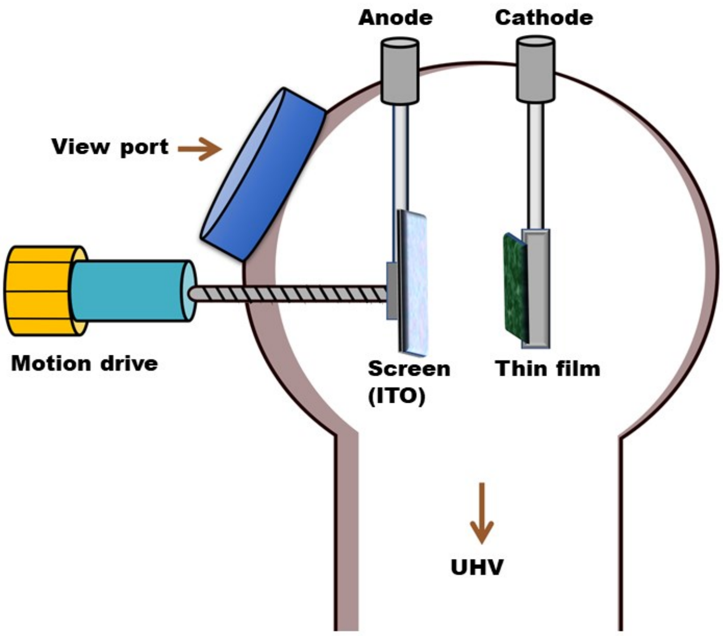

Supplement: RA-010-D0RA03360J-s001 [file RA-010-D0RA03360J-s001.pdf]
